# A LTCC-Based Ku-Band 8-Channel T/R Module Integrated with Drive Amplification and 7-Bit True-Time-Delay

**DOI:** 10.3390/s22176568

**Published:** 2022-08-31

**Authors:** Xiao Liu, Qinghua Zeng, Zhengzhi Ding, Haitao Xu

**Affiliations:** 1 School of Electronic, Electrical and Communication Engineering, University of Chinese Academy of Sciences, Beijing 100049, China; 2 Aerospace Information Research Institute, Chinese Academy of Sciences, Beijing 100094, China; 3 Department of Communication Engineering, University of Science and Technology Beijing, Beijing 100083, China

**Keywords:** Ku-band, T/R module, true-time-delay, phased array

## Abstract

Ku-band drive amplification and a 7-bit true-time-delay (TTD) function were realized as a part of a LTCC-based T/R module to increase integration. The 8-channel T/R module was fabricated and its key characteristics were measured, including a 3-bit (1/2/4 λ) TTD, 4-bit (0.25/0.5/1/2 λ) TTD, receive gain, noise figure and output power. The 8-channel T/R module can be further adopted to increase bandwidth and scanning angle of phased arrays without beam squint.

## 1. Introduction

Active electronically scanned arrays (AESA) with a number of transmit/receive (T/R) modules are widely adopted due to the rapid change of beam shape and beam direction [1,2,3]. Phase shifters are used to adjust the phase of each element and yet beam squint still occurs for wide-band applications [4,5,6,7]. Therefore, true-time-delay (TTD) modules are essential in phased array systems such as AESA radars to meet the increasing demand of broader bandwidth and wider scanning angle [8,9,10,11].

Fenn et al., reported a 300–450 MHz dipole linear array antenna and TTDs can be implemented by using appropriate lengths of coaxial cable transmission lines in order to maintain the main beam pointed at a fixed 40-degree angle from broadside over the operating bandwidth [12]. However, more and more functions are designed to be combined with TTD function to meet the increasing demand of higher integration density and smaller module size. Liu et al., reported an X-band receiver integrated with a subarray-level 5-bit TTD module [13]. Shi et al., reported a Ka-band high integration receiver module which combines TTD units at sub-array level and phase shifters at array-element level to realize a 1.6 GHz instantaneous bandwidth [14]. Tang et al. reported an X-band 5-bit time-delay amplifier module in which a time-delay function is realized by coplanar waveguide (CPW) transmission lines [15]. Guo et al. reported a 6-bit TTD amplifier module for X-band subarray phased arrays where CPW transmission lines are also used as time delay units [16]. Li et al., reported a C-band subarray drive time-delay module which integrates 5-bit time-delay, amplification and power dividing/combining [17,18]. Li et al., reported an X-band subarray drive time-delay module which integrates 5-bit time-delay, and receives and transmits gain compensation [19]. In both modules, microstrip lines and striplines with different lengths are used to realize time-delay function. However, using CPW transmission lines or microstrip lines and striplines as delay paths results in an unacceptable amount of module area for the required time delay, and the insertion loss of different time delay states varies substantially [20,21].

In terms of electrical design and micro/macro-packaging fabrication, electronic delay lines using monolithic microwave integrated circuit (MMIC) devices can be more easily implemented through multi-chip module (MCM) technology for higher integration and smaller circuit diagrams [22,23,24,25]. Zhang et al., reported a C-band multi-channel 9-bit TTD amplification module, and GaAs MMICs are adopted to realize TTD function by switching between a reference path and a delay path [26,27]. However, on the one hand, extra bi-directional amplifiers are needed to compensate the signal transmission loss induced by insertion loss of TTD MMICs [28]. Not only TTD units but also amplifiers are integrated in one active MMIC device to combat insertion losses and to provide gain [29,30]. On the other hand, it is barely reported that TTD modules and T/R modules are integrated as a whole. Integrating a TTD module with a T/R module can greatly improve the packaging density and simplify the interconnection among modules inside a complicated antenna system.

In this paper, a LTCC-based Ku-band TTD module is integrated with a Ku-band T/R module inside the same shell. LTCC technology is very suitable for the manufacturing of mixed-signal multi-layer substrates, which can realize the combined design of microwave circuits, logic control circuits and power management circuits on different layers. The TTD module integrates a 7-bit time-delay, receive gain compensation and transmit power drive, which are realized through active MMICs.

The paper is organized as follows: the topography and link budget of the 8-channel T/R module with 7-bit TTD are presented in Section 2. Section 3 reports the simulation results of the SMP-microstrip vertical transition and strip line power divider for the Ku-band. Measurements of the 8-channel T/R module with 7-bit TTD are reported in Section 4 and conclusions are provided in Section 5.

## 2. Topography and Link Budget of the 8-Channel T/R Module with 7-Bit TTD

**Figure 1:**
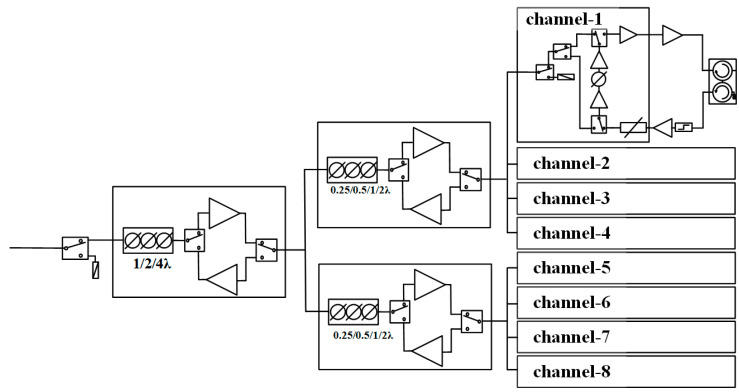
Topography of the 8-channel T/R module with 7-bit TTD.

**Figure 2:**
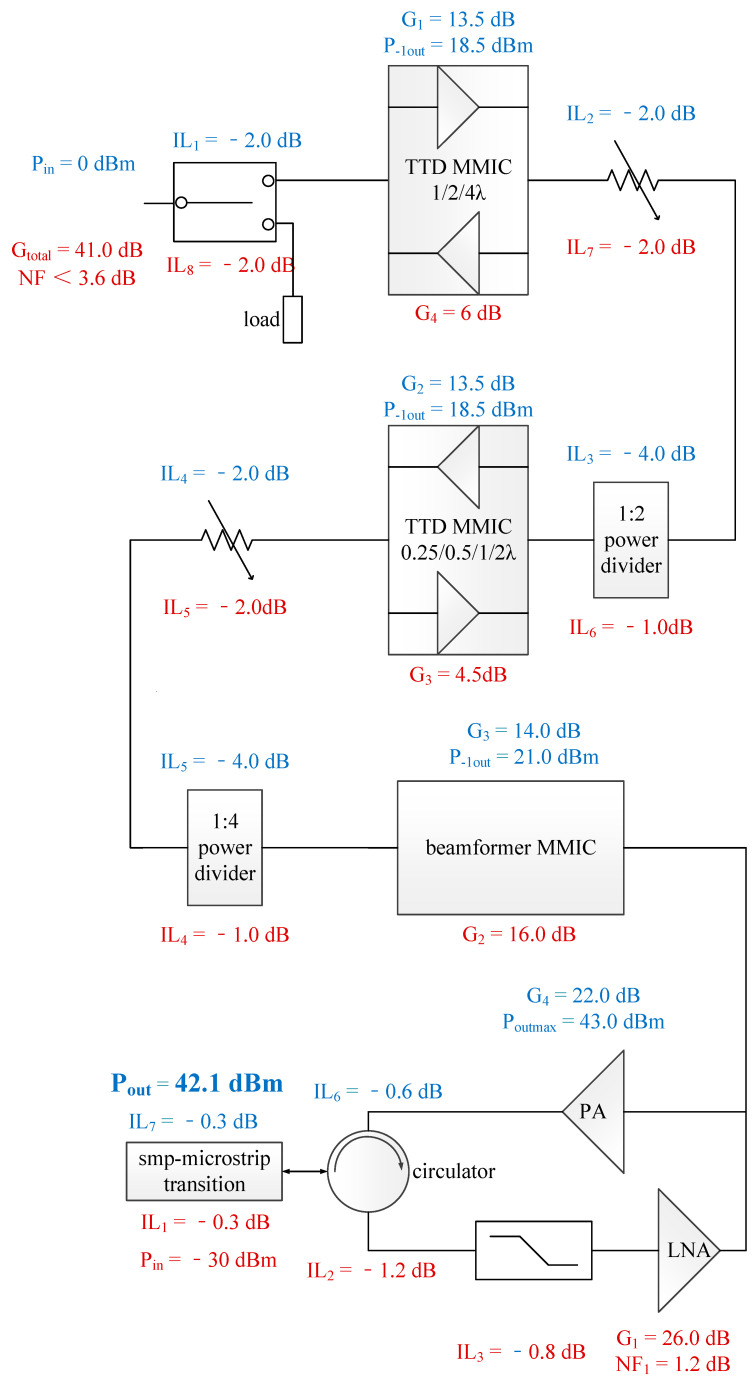
Link budget of receive (red) and transmit (blue).

As shown in Figure 1 and Figure 2, a 4-bit (0.25/0.5/1/2 λ) TTD MMIC (*f*_0_ = 16.7 GHz and *t*_delay_ = 15 ps) is used for every four channels and a 3-bit (1/2/4 λ) TTD MMIC (*f*_0_ = 16.7 GHz and *t*_delay_ = 60 ps) is used for every eight channels. Not only TTD function but also receive gain compensation, transmit power drive and switches are all integrated in TTD MMICs.

Each channel mainly consists of a GaAs beamformer MMIC, a GaAs low noise amplifier (LNA), a GaAs limiter, a GaN power amplifier (PA), a circulator, a pulse modulation circuit and a power supply circuit. Amplifiers, phase shifters, attenuators and serial-to-parallel converters are integrated in the beamformer MMIC. A 2-way power divider and two 4-way power dividers are realized through LTCC-based striplines. The circulators used in this module are three-port ferrite circulators. Under transmit mode, a wave incident on port 1 is coupled into port 2; under receive mode, a wave incident on port 2 is coupled into port 3. The typical isolation S32 is more than 27 dB.

Under receive mode, the theoretical net gain is 41 dB and the noise figure (NF) is less than 3.6 dB. Under transmit mode, the maximum output power is 42.1 dBm (16 W).

**Figure 3:**
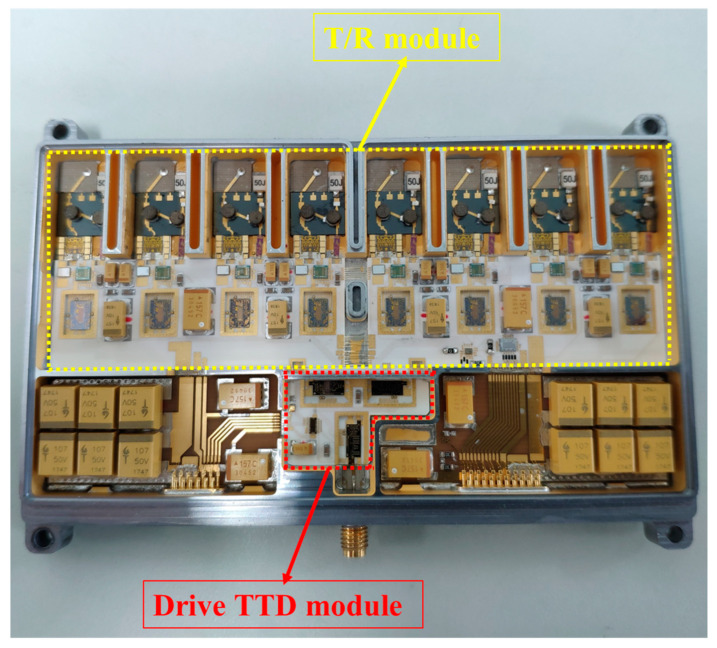
A photo of the 8-channel T/R module with 7-bit TTD.

Increased integration density and decreased weight are achieved with 15 layers of stacked LTCC substrates and a 50AlSi shell. High power GaN PAs are welded to molybdenum copper substrates and then sintered on 50AlSi shells to improve heat dissipation. The fabricated 8-channel T/R module is shown in Figure 3. The size is 110 mm × 65 mm × 10 mm and the weight is 85 g.

## 3. Simulation of SMP-Microstrip Vertical Transition and Stripline Power Divider for Ku-Band

**Figure 4:**
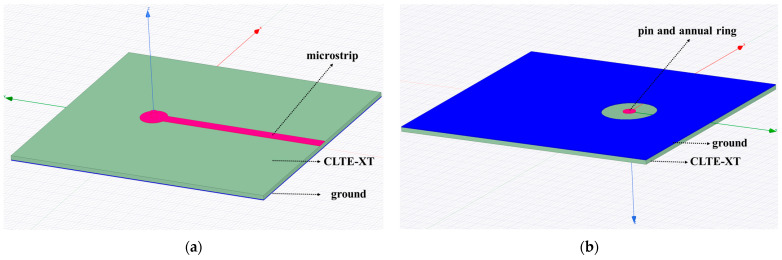
SMP-to-microstrip vertical transition model: (**a**) front side of the simulation model; (**b**) back side of the simulation model.

In this module, a vertical transition of SMP-to-microstrip is adopted, as illustrated in Figure 4. The SMP pin passes through the CLTE-XT substrate with a metallized via. Simulation was carried out to optimize the three critical parameters: the radius of the metallized via, the radius of the annular ring and the width between the annular ring and the ground, and the width of the microstrip line.

**Figure 5:**
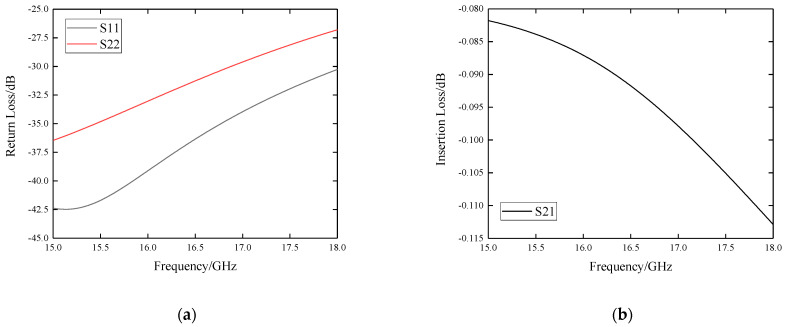
Simulation results of the SMP-to-microstrip vertical transition. (**a**) Return loss S11 and S22; (**b**) Insertion loss S21.

Simulation results of S11, S22 and S21 are shown in Figure 5. The return loss is below −25 dB and the insertion loss is below −0.15 dB.

**Figure 6:**
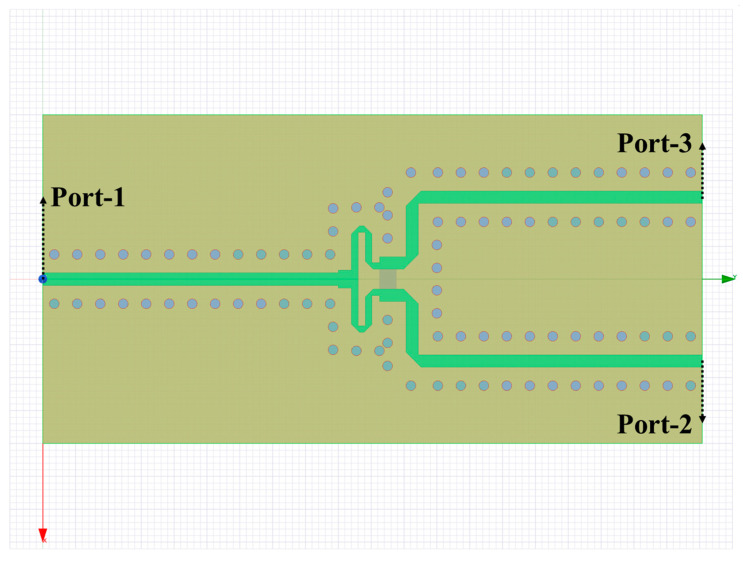
LTCC-based stripline 2-way power divider for Ku-band.

LTCC substrates are adopted for the T/R modules and TTD modules. A 2-way Wilkinson power divider and a 4-way Wilkinson power divider are realized by means of LTCC-based stripline to further increase integration. The simulation model of the LTCC-based stripline 2-way power divider is shown in Figure 6. Alongside the stripline, grounding vias are adopted to constrain the transmission of the electromagnetic wave to decrease the loss of electromagnetic energy. Based on the 2-way power divider, the 4-way power divider is realized through a cascading two-level 2-way power divider.

**Figure 7:**
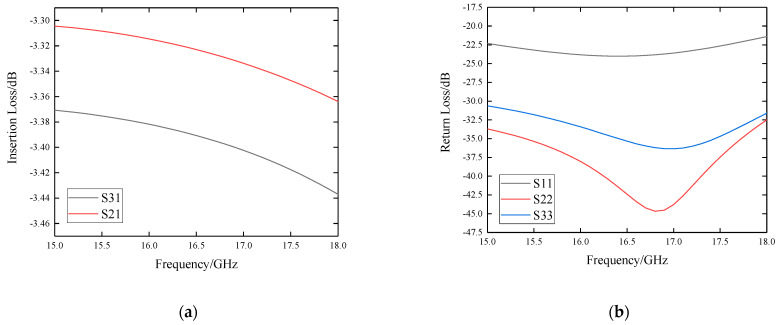

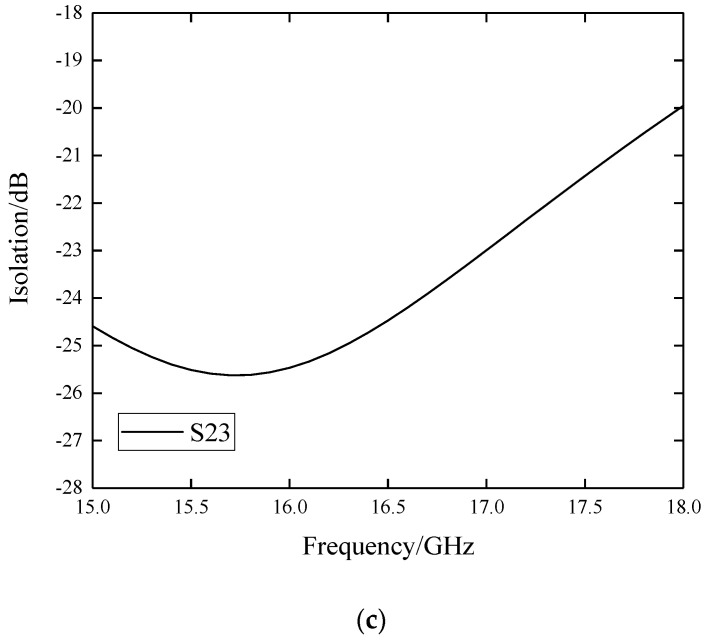
Simulation results of the LTCC-based stripline 2-way power divider: (**a**) insertion loss S21 and S31; (**b**) return loss S11, S22 and S33; (**c**) isolation S23.

The simulation results are shown in Figure 7. The insertion loss is below −3.5 dB and the difference between that of port-2 and port-3 is less than 0.1 dB. The return loss of port-1 is less than −20 dB and the return loss of port-2 and port-3 is less than −30 dB. The isolation between port-2 and port-3 is less than −20 dB.

## 4. Measurement of the 8-Channel T/R Module with 7-Bit TTD

Measurement of the 8-channel T/R module with 7-bit TTD includes TTD, NF, receive gain and output power.

### 4.1. TTD

The TTD units in active MMICs are basically composed of SPDT switches, DPDT switches and time delay elements such as CLC π-networks. TTD is calculated by the unwrapped phase, which was measured through Agilent vector network analyzer N5244A.

Firstly, zero-centering is carried out based on the unwrapped phase of the middle frequency, as shown in Equation (1).

(1) $$P\left(f\right)=P\left(f_{x}\right)-P\left(f_{0}\right),$$

$P\left(f_{0}\right)$ is the unwrapped phase of the middle frequency and $P\left(f_{x}\right)$ is the unwrapped phase of each frequency within the bandwidth.

Secondly, the delay wavelength of each frequency $n_{x}\;$ is calculated based on the middle frequency, as shown in Equation (2).

(2) $$n_{x}=P\left(f\right)/\left[360\times \left(1-f_{x}/f_{0}\right)\right],$$

Thirdly, the least square method is adopted to fit $n_{x}$ and the fitted *n* is further used to calculate the TTD value ∆P(f), as shown in Equation (3).

(3) $$∆P\left(f\right)=P\left(f\right)-n\times 360\times \left(1-f_{x}/f_{0}\right),$$

Measured TTD and the error with a theoretical value are listed in Table 1. For the 4-bit TTD, the maximum relative error is approximately 15%. For the 3-bit TTD, the maximum relative error is approximately 10%.

### 4.2. Noise Figure

**Figure 8:**
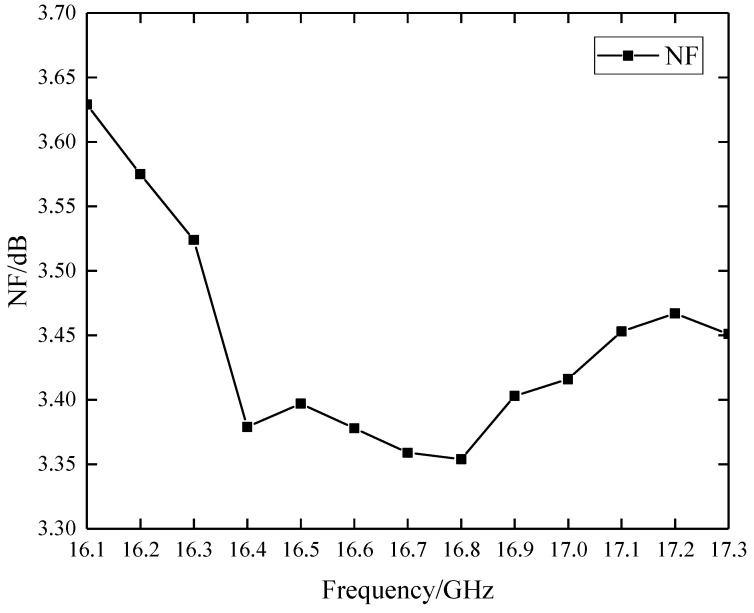
Measured results of NF.

NF was measured using the Agilent Noise Figure Analyzer N8975A. As shown in Figure 8, the measured results of NF are below 3.65 dB within the bandwidth of 1.2 GHz, which meets the theoretical value of 3.6 dB.

### 4.3. Receive Gain

The characteristics of receive were measured using the Agilent N5244A Network Analyzer.

**Figure 9:**
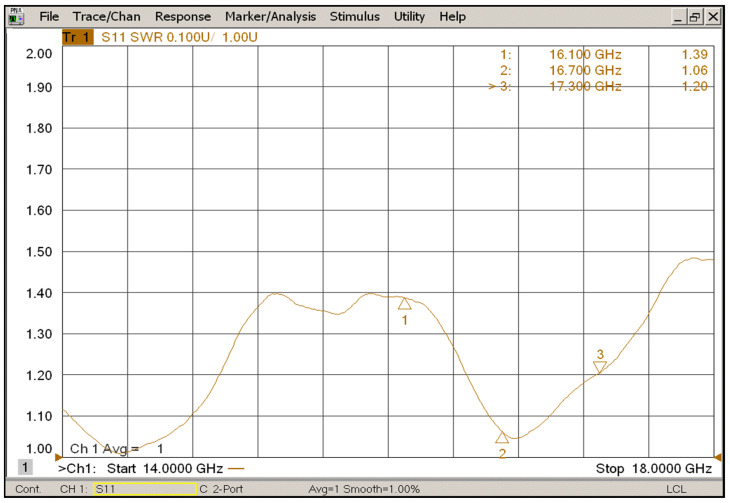
Measured SWR of the receive input port.

The standing wave ratio (SWR) of the receive input port is shown in Figure 9. The SWR within the bandwidth (from 16.1 GHz to 17.3 GHz) is less than 1.4.

**Figure 10:**
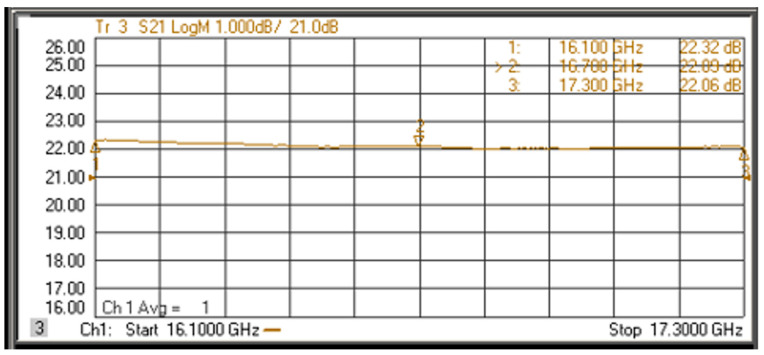
Measured results of receive.

The minimum output power of the network analyzer is −30 dBm and a 10 dB attenuator is added in front of the receive input port so that the whole module works in the linear region. The measurements of receive within the bandwidth of 1.2 GHz are shown in Figure 10. S21 is approximately 22 dB with a difference within the bandwidth of less than 0.5 dB. The net gain of receive is 22 + 10 (10 dB attenuator) + 9 (4-way and 2-way divider) = 41 dB.

### 4.4. Output Power

Under transmit mode, the output power was measured using the Agilent N1912A Power Meter. Within the bandwidth of 1.2 GHz, GaN power amplifiers can work under saturation mode with input power varying from −2 dBm to 2 dBm. The maximum duration for transmit is 100 microseconds with a duty ratio of 10%. The measured output power is larger than 42.3 dBm (17 W).

**Figure 11:**
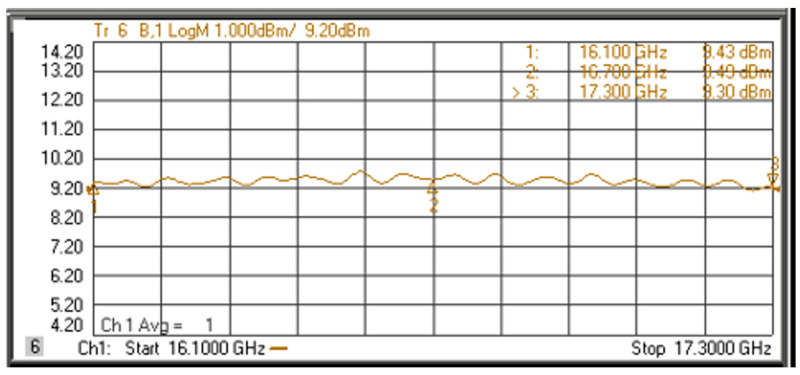
Measured results of transmit.

The measurements of transmit through the network analyzer are shown in Figure 11. A 30 dB attenuator is added in front of port 2 to protect the network analyzer from overpower. The insertion loss of the cable for measurement is about 3 dB.

The measured output power is between 9.12 dBm and 9.76 dBm, with a difference of less than 0.7 dB.

## 5. Conclusions

A Ku-band 8-channel T/R module with 7-bit TTD function was designed and fabricated. LTCC-integrated power dividers and vertical transmission of SMP-to-microstrip were designed and simulated. Each state of the 7-bit TTD was measured and the relative error was less than 15.2%. Under receive mode, the net gain was 41 dB with a difference within the bandwidth of 1.2 GHz of less than 0.5 dB. The measured NF was less than 3.65 dB. Under transmit mode, the output power was larger than 42.3 dBm (17 W) with a difference of less than 0.7 dB.

## Figures and Tables

**Table 1:** Measurement results of 7-bit TTD and accuracy of each state.

Theoretical TTD/λ	Measured TTD/λ	Absolute Error/λ	Relative Error/%
0.25 (of 4-bit TTD)	0.2880	0.0380	15.20%
0.5 (of 4-bit TTD)	0.4957	0.0043	0.86%
1 (of 4-bit TTD)	0.9565	0.0435	4.35%
2 (of 4-bit TTD)	2.0057	0.0057	0.29%
1 (of 3-bit TTD)	0.8954	0.1046	10.46%
2 (of 3-bit TTD)	2.0998	0.0998	4.99%
4 (of 3-bit TTD)	4.1074	0.1074	2.69%

## Data Availability

Not applicable.
